# The Spread of Tomato Yellow Leaf Curl Virus from the Middle East to the World

**DOI:** 10.1371/journal.ppat.1001164

**Published:** 2010-10-28

**Authors:** Pierre Lefeuvre, Darren P. Martin, Gordon Harkins, Philippe Lemey, Alistair J. A. Gray, Sandra Meredith, Francisco Lakay, Adérito Monjane, Jean-Michel Lett, Arvind Varsani, Jahangir Heydarnejad

**Affiliations:** 1 Institute of Infectious Diseases and Molecular Medicine, University of Cape Town, Observatory, Cape Town, South Africa; 2 CIRAD, UMR 53 PVBMT CIRAD-Université de la Réunion, Pôle de Protection des Plantes, Ligne Paradis, Saint Pierre, La Réunion, France; 3 Centre for High-Performance Computing, Rosebank, Cape Town, South Africa; 4 South African National Bioinformatics Institute, University of the Western Cape, Cape Town, South Africa; 5 Department of Microbiology and Immunology, Katholieke Universiteit Leuven, Leuven, Belgium; 6 Electron Microscope Unit, University of Cape Town, Rondebosch, Cape Town, South Africa; 7 Department of Molecular and Cell Biology, University of Cape Town, Rondebosch, Cape Town, South Africa; 8 School of Biological Sciences, University of Canterbury, Christchurch, New Zealand; 9 Department of Plant Protection, College of Agriculture, Shahid Bahonar University of Kerman, Kerman, Iran; ILTAB/Donald Danforth Plant Science Center, United States of America

## Abstract

The ongoing global spread of Tomato yellow leaf curl virus (TYLCV; Genus *Begomovirus*, Family *Geminiviridae*) represents a serious looming threat to tomato production in all temperate parts of the world. Whereas determining where and when TYLCV movements have occurred could help curtail its spread and prevent future movements of related viruses, determining the consequences of past TYLCV movements could reveal the ecological and economic risks associated with similar viral invasions. Towards this end we applied Bayesian phylogeographic inference and recombination analyses to available TYLCV sequences (including those of 15 new Iranian full TYLCV genomes) and reconstructed a plausible history of TYLCV's diversification and movements throughout the world. In agreement with historical accounts, our results suggest that the first TYLCVs most probably arose somewhere in the Middle East between the 1930s and 1950s (with 95% highest probability density intervals 1905–1972) and that the global spread of TYLCV only began in the 1980s after the evolution of the TYLCV-Mld and -IL strains. Despite the global distribution of TYLCV we found no convincing evidence anywhere other than the Middle East and the Western Mediterranean of epidemiologically relevant TYLCV variants arising through recombination. Although the region around Iran is both the center of present day TYLCV diversity and the site of the most intensive ongoing TYLCV evolution, the evidence indicates that the region is epidemiologically isolated, which suggests that novel TYLCV variants found there are probably not direct global threats. We instead identify the Mediterranean basin as the main launch-pad of global TYLCV movements.

## Author Summary

Tomato yellow leaf curl virus (TYLCV) poses a serious threat to tomato production throughout the temperate regions of the world. Our analysis, using a suite of bioinformatic tools applied to all publically available TYLCV genome sequences, suggests that the virus probably arose somewhere in the Middle East between the 1930s and 1950s and that its global spread only began in the 1980s after the emergence of two strains – TYLCV-Mld and -IL. In agreement with others, we also find that the highly invasive TYLCV-IL strain has jumped at least twice to the Americas – once from the Mediterranean basin in the early 1990s and once from Asia in the early 2000s. Although our results corroborate historical accounts of TYLCV-like symptoms in tomato crops in the Jordan Valley in the late 1920s, they indicate that the region around Iran is both the current center of TYLCV diversity and is the site where the most intensive ongoing TYLCV evolution is taking place. However, our analysis indicates that this region is epidemiologically isolated suggesting that novel TYLCV variants found there are probably not direct global threats. Moreover, we identify the Mediterranean basin as the main launch-pad of global TYLCV movements.

## Introduction

Tomato yellow leaf curl disease (TYLCD) is one of the most devastating emerging diseases of tomato in the warm and temperate regions of the world. It is caused by a complex of at least six virus species in the *Begomovirus* genus of the Family *Geminiviridae* [1], [2]. Tomato yellow leaf curl virus (TYLCV) is the most widely distributed and best studied of these species and the begomoviruses responsible for TYLCD are therefore collectively referred to as TYLCV-like viruses. Besides TYLCV, the TYLCV-like viruses include Tomato yellow leaf curl Sudan virus (TYLCSDV), Tomato yellow leaf curl Axarquia virus (TYLCAxV), Tomato yellow leaf curl Malaga virus (TYLCMLV), Tomato yellow leaf curl Sardinia virus (TYLCSV) and Tomato yellow leaf curl Mali virus (TYLCMLV) [1], [3].

Although TYLCV-like viruses were first described in the Jordan Valley in Israel during the early 1960s, disease symptoms resembling TYLCD (stunted tomato plants with downward leaf curling, leaf discoloration and leaf deformation) had been observed in the Jordan Valley since the late 1920s (cited in [4], [5]). In Israel during the early 1990s, two begomovirus strains associated with TYLCD infections of different severities were cloned and named Tomato yellow leaf curl virus–Israel (TYLCV-IL; TYLCV-IL[IL:Reo:86]-X15656) and Tomato yellow leaf curl virus–Mild (TYLCV-Mld; TYLCV-Mld[IL:93] – X76319) [5], [6]. It was subsequently determined that TYLCV-IL was a recombinant of the TYLCV-Mld strain and another begomovirus species related to Tomato leaf curl Karnataka virus (ToLCKV; [7]). TYLCV-IL contains mostly TYLCV-Mld like sequences but the 5′-portion of its *rep* gene is very ToLCKV-like. Other subsequently characterised TYLCV strains such as the Gezira (e.g. TYLCV-Gez[SD:96]), Iran (e.g. TYLCV-IR[IR:Ira:98]) and Oman (e.g. TYLCV-OM[Om:Alb:05]) also display evidence of having arisen through unique, albeit similar, inter-species recombination events [8]–[10].

Of all the known TYLCV strains, TYLCV-IL and TYLCV-Mld have the broadest geographical ranges stretching in the Old world from Japan in the east [11] to Spain in the west [12] and the Indian Ocean island of Reunion [13] and Australia [14] in the south. Additionally, TYLCV-IL has apparently jumped at least twice between the Old and New Worlds [15], [16] and is currently spreading into North and South America [17]–[19]. As the international trafficking of crop varieties is relatively widespread, it is perhaps not surprising that a virus like TYLCV-IL could attain such a global distribution. Nevertheless, amongst the geminiviruses, the TYLCV-IL geographical range is unusually vast.

Given that the Mediterranean basin and the Middle East are clearly centers of TYLCV diversity [20], it is probable that this is where these viruses originate. The region has a climate that favors tomato cultivation and collectively accounts for 30% of global tomato production (FAOSTAT 2008). It is of some concern therefore that recent reports have indicated a dramatic increase in TYLCD incidence within the region [10], [21]–[24]. In Iran in particular where the climate has warmed and dried in recent years there has apparently been a steady increase in the incidence of whitefly transmitted geminivirus diseases in tomato crops [9], [25]–[29].

Considering the high degrees of TYLCV diversity in the Middle East and the amount of inter-strain and inter-species recombination that has been detected between TYLCV and different Middle Eastern begomovirus species [9], [10], it is reasonable to suspect that virus evolution within this region has had, and will probably continue to have, a major impact on global TYLCD epidemiology. We therefore isolated and sequenced 15 new Iranian TYLCV isolates which were used along with publicly available sequences both to identify where TYLCV originated, and to retrace the virus' movement patterns around the globe. Together with detailed recombination analysis, we applied a newly developed Bayesian phylogeography method to infer where and when major events in the evolution of TYLCVs have occurred. In congruence with previous assumptions, our analysis clearly indicates both that the emergence and global spread of TYLCV have been extremely rapid, and that the Middle East in general, and the region surrounding Iran in particular, are probably the current and past centers of ongoing TYLCV diversification.

## Materials and Methods

### Sampling and DNA extraction

Samples from 27 tomato plants displaying typical TYLCD symptoms (upward leaf curling, yellowing, distortion, and stunting) were collected in the major tomato producing regions of Southern Iran (Kerman, Hormozgan, Bushehr and Fars provinces) in 2006 and 2007 (Table S1). Total DNA was extracted from the fresh or dried leaves using High Pure Viral Nucleic Acid Extraction Kit (Roche, Germany) according to the method described by the manufacturer.

### Isolation, cloning and sequencing of full length genomes

DNA-B and DNAβ molecules that are commonly found within begomovirus infections were tested for using the primer pairs PBL1v2040/PCRc1 [30], and Beta01/Beta02 [31].

Viral genomes were amplified from total plant DNA extractions using phi29DNA polymerase (TempliPhi, GE Healthcare, USA) as previously described [32], [33]. Amplified genomic concatemers were digested with either *Xmn*I or *Pst*I to yield full length genomes (∼2.7 kb). The linearised fragments were either ligated to *Pst*I digested pGEM 3Zf+ (Promega Biotech) or blunt-end ligated to the blunt cloning site of pJET1.2 (CloneJET PCR cloning kit, Fermentas). Full genomes were commercially sequenced (Macrogen Inc., Korea) on both strands by primer walking. Sequences were assembled and edited using dnaman (version 5.2.9; Lynnon Biosoft) and MEGA 4 [34].

### Phylogenetic and recombination analyses

The 15 new TYLCV genomes were aligned with all full-length begomoviruses, DNA-A and DNA-A-like sequences available in GenBank in July 2009 using POA v2 [35]. This alignment was edited by eye in MEGA 4 [34] with ∼595 poorly aligned alignment columns within the intergenic region being removed from all subsequent analyses (the resulting alignment is available on request from the authors). Maximum likelihood phylogenetic trees were constructed with PHYML [36] with model GTR+G_4_ (selected as the best-fitting model by RDP3; [37] and 1000 full maximum likelihood (ML) bootstrap iterations. Degrees of sequence identity shared by sequences were calculated using MEGA 4 with pairwise deletion of gaps.

Detection of potential recombinant sequences, identification of likely parental sequences, and localisation of possible recombination breakpoints was carried out on using the RDP [38], GENECONV [39], BOOTSCAN [40], MAXIMUM CHI SQUARE [41], CHIMAERA [37], SISCAN [42] and 3SEQ [43] recombination detection methods as implemented in RDP3 [37]. The analysis was performed with default settings for the different detection methods and a Bonferroni corrected *P*-value cut-off of 0.05. Only events detected with two or more methods coupled with significant phylogenetic support were considered credible evidence of recombination. The breakpoint positions and recombinant sequence(s) inferred for every detected potential recombination event were manually checked and adjusted where necessary using the extensive phylogenetic and recombination signal analysis features available in RDP3.

### Phylogeographic analysis and evolutionary rate estimation

The movement patterns of TYLCV over the past century were reconstructed using a recently developed approach that, given a set of sequences sampled from various discreet locations (such as individual cities, countries or other geographical regions) over a few decades, models changes in geographical location during the evolution of the sequences [44]. This fully probabilistic approach, implemented in the computer program, BEAST v1.5.3 [44], draws on an explicit model describing how, during the evolution of the sampled sequences since their last common ancestor, the unknown geographical locations of ancestral sequences have changed between the known locations of these sampled sequences. In a process that is very similar to that used to infer ancestral nucleotide sequences, the methodology employs continuous-time Markov chain models of discrete state evolution (meaning that rather than the individual GPS coordinates of each sequence being considered, all the sequences from the same approximate region are assigned the same region state) to determine the most probable geographical locations of ancestral sequences. Besides inferring where amongst the sampling locations ancestral sequences most likely resided, the method additionally provides a statistically meaningful measure of the over-all confidence that can be associated with movements between any two of these locations. This is achieved by using a so-called Bayesian stochastic search variable (BSSV) procedure [44] which is associated with a Bayes factor [45], [46] test that can be used to identify the best supported movement routes between the various geographical locations considered.

Following the results of Duffy and Holmes [47] we assumed a constant population size tree prior and a log-normal relaxed molecular clock for our TYLCV phylogeographic analyses. Individual BEAST runs were performed with 200 million steps in the Markov chain and sampling every 10,000 steps to produce a posterior tree distribution containing 20,000 genealogies. Similar results allowed us to combine log and tree files using LogCombiner (available in BEAST package). The maximum clade credibility tree (a point estimate of the tree with the highest cumulative posterior probabilities in the posterior distribution of trees) was annotated with geographical locations using the software TreeAnnotator (available in BEAST package).

We used tools available from http://beast.bio.ed.ac.uk/Google_Earth to produce a graphical animation in key markup language (kml) file format of the spatio-temporal movement dynamics of ancestral TYLCV sequences. These kml files, available as Dataset S1 and Dataset S2, contain information on routes and times of virus movements can be viewed using Google Earth (available from http://earth.google.com).

Two temporally structured TYLCV datasets (sampling dates spanning from 1988 to 2009) were analysed (see Table S1 for details). Whereas the first, contained 82 full TYLCV genomes and was called the FG dataset, the second contained 91 ∼940 nt long TYLCV sequences corresponding to genome positions 148–1090 in isolate TYLCV-IL[IL:Reo:86] (accession number X15656) and was called the CP dataset. While the FG dataset contained substantial evidence of inter-species recombination (particularly in the sequences encoding the complementary sense genes), the CP dataset was mostly free of detectable recombination and contained absolutely no evidence of inter-species recombination. Therefore, although it contained fewer phylogenetically informative sites, analyses of the CP dataset were expected to be free of the confounding effects that recombination in the FG dataset might have on estimates of substitution rates and sequence divergence times [48], [49]. Using the sampling coordinates and a freely available hierarchical clustering method (called “hclust”) implemented in R [50], we were able to optimally define groups of sequences displaying definite geographical clustering. Longitude and latitude coordinates at the centroids of each of the groups thus defined, were used as the discrete sampling locations in our phylogeographic analyses. The sequences in the FG and CP datasets were respectively grouped into seven and nine of these discreet sampling locations (see Table S1 for details). It is important to stress that despite the fact that the dendrogram constructed during the geographical clustering analysis superficially resembles a phylogenetic tree, the groupings depicted by the dendrogram are based entirely on relative geographical proximity and not on relative sequence similarity and as a result the clustering methods could have in no way confounded our subsequent phylogeographic analyses.

Whereas for the FG dataset similar numbers of sequences (between 8–24) were sampled from the various locations considered (the exceptions are Reunion and the Horn of Africa with only 2 and 1 samples respectively), there were quite significant sampling biases in the CP dataset with substantially more sequences having been sampled from Iran (∼33%) relative to the other locations considered. We used two separate tests to assess the consequences of such sampling biases on our analyses. In the first test we “equalised” the sample sizes for all locations from which more than eight sequences had been sampled by randomly sub-sampling eight sequences from each of these. For each of ten smaller datasets thus constructed from both of the FG and CP datasets (the FG-based datasets contained 51 sequences and the CP-based datasets 42 sequences) we performed the same phylogeographic analyses as those described above. In the second test, the analysis was also carried out as above but the location states of the sequences were randomized using an additional operator in the MCMC procedure (BEAST can be set up to do this). The location state probabilities of the root node determined during these analyses were compared with those determined for the datasets analysed without the location state randomization setting.

### Dating and locating ancestral recombinants

Based on the dated maximum clade credibility (MCC) trees constructed from the temporally structured FG and CP datasets and the parental and recombinant sequences identified in our recombination analyses we could determine the approximate dates when recombination events occurred and pinpoint the geographical locations of the ancestral recombinants. For each detected recombination event we first constructed a neighbour joining tree based on the TYLCV derived sequences found within the recombinant (using a Jukes Cantor nucleotide substitution model in RDP3). The date ascribed to the corresponding node in the dated MCC tree that marked the branching point of the recombinant sequence(s) (in many cases there were multiple sequences descended from a single ancestral recombinant) was taken to be the earliest date when the recombination event could have occurred (with the earlier bound of the associated 95% highest probability density, or HPD, indicating the lowest credible bound of this estimate). This “lower” node essentially represents the most recent common ancestor of the recombinant(s) with a non-recombinant. In cases where multiple sequences appeared to bare traces of the same ancestral recombination event, the date associated with the MCC tree node representing the last common ancestor of the recombinant sequences was taken as being the latest probable date when the recombination event might have occurred (with the upper bound of the associated 95% HPD indicating the upper credible bound of this estimate). This “upper” node represents the most recent common ancestor of the recombinants. To determine the approximate geographical location of where recombination events might have occurred the inferred geographical locations of sequences at these “lower” and “upper” nodes were assumed to bound the location where the recombination event in question occurred. In cases where only a single sequence carried evidence of a recombination event, the latest date of the recombination event and the upper bound of the 95% HPD of this date were taken as the sampling date of the sequence. In such cases the “upper” bound on the geographical location where the recombination event may have occurred was simply taken to be the sampling location of the recombinant.

## Results/Discussion

### Iran is a center of TYLCV diversity

**Figure 1:**
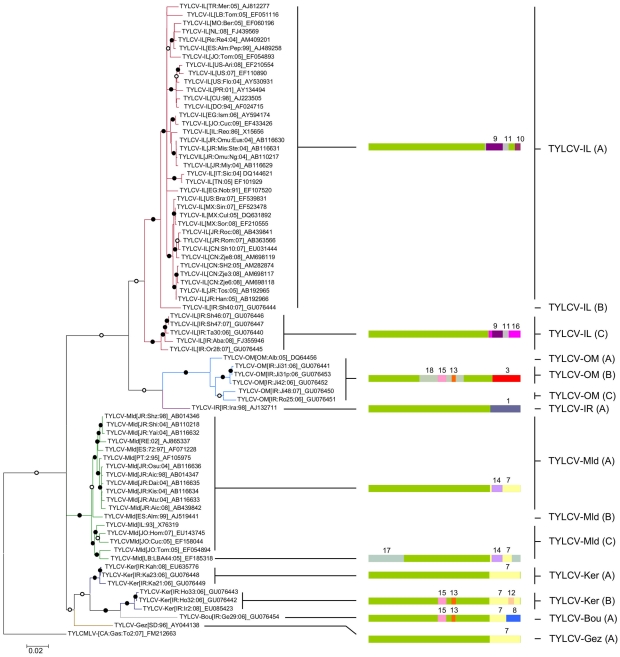
Maximum likelihood phylogenetic tree (constructed with GTR+G_4_ selected as the best fit model by RDP3 and rooted using a tomato yellow leaf curl Mali virus, or TYLCMV, isolate) depicting the relatedness of representative TYLCV full genome sequences. While branches supported in >90% of bootstrap replicates are marked with filled circles, those supported in >70% of replicates are marked with open circles, and those supported in <50% of replicates have been collapsed. Twelve unique recombination events yielding ten different recombination patterns are presented to the right of the tree. Whereas green colours indicate TYLCV derived sequences all other colours indicate sequences derived from non-TYLCV sources. Recombination events are numbered according to Table 1.

We collected samples showing TYLCD symptoms in the provinces of Kerman (Kahnooj, n = 4; Jiroft, n = 5 Orzuiyeh, n = 1), Fars (Shiraz, n = 6; Lar, n = 1), Yazd (Taft, n = 1; Ashkezar, n = 1), Hormozgan (Roodan, n = 4; Minab, n = 3) and Bushehr (Borazjan, n = 1) and cloned and determined full-length DNA-A-like sequence from 15 of these (Kahnooj, n = 2; Jiroft, n = 4; Orzu'iyeh, n = 1; Shiraz, n = 3; Taft, n = 1; Roodan, n = 1; Minab, n = 2; Borazjan = 1). No DNA-B or Beta molecules were detected in any of the analysed samples. Phylogenetic analysis and pairwise genome-wide similarity comparisons between these 15 new sequences and those deposited in sequence databases (Figure 1 and Figure S1) indicated that five were TYLCV-IL isolates, five were TYLCV-OM isolates, four were TYLCV-Ker isolates and one was an isolate of a potentially new strain that we have tentatively named TYLCV-Bou. TYLCV-Bou represents a new strain based on the currently accepted geminivirus strain demarcation criteria [3] in that it shares 92.5–94% identity with TYLCV-Ker isolates (Figure S1). Different isolates from the individual strain groupings displayed minimal evidence of geographical clustering within Iran (see Figure S2).

**Table 1:** Recombination events detected in TYLCV sequences.

Event	Recombinant(s)	Non-TYLCV Parent	TYLCV Parent	Breakpoints^1^		Methods	P-value	Date of Origin				Place of Origin							
												FG				CP			
								FG		CP		Rec Anc		Non-rec Anc		Rec Anc		Non-rec Anc	
				Begin	End			upper	lower	upper	lower	Location	p-val	Location	p-val	Location	p-val	Location	p-val
1	TYLCV-IR[IR:Ira:98]	ToLCIRV	TYLCV-Mld/-IL	1	2131	RGBMCST	2.06E-77	1996	1919	1996	1985	Iran	1	Iran	0.96	Iran	1	Israel	0.59
2	TYLCAxV	TYLCSV	TYLCV-IL (A)	1702	411	RGBMCST	3.42E-90	2000	1995	2000	1989	W Med	1	Reunion	0.51	W Med	1	W Med	1
3	TYLCV-OM	ToLCKV	TYLCV-Mld/-IL	2145	396	RGBMCST	5.15E-61	1981	1968	2000	1996	Iran	1	Iran	0.99	Iran	1	Iran	1
4	TYLCMalV	TYLCSV	TYLCV-Mld (A)	407	1110*	RGBMST	1.91E-59	1999	1975	1999	1994	W Med	1	W Med	0.82	W Med	1	W Med	1
5	TYLCAxV-Sic1-[IT:Sic2/2:04	TYLCSV	TYLCV-IL (A)	423	2000*	RGBMCST	1.67E-53	2004	1971	2004	1987	W Med	1	W Med	0.51	W Med	1	W Med	0.55
6	TYLCAxV-Sic2-[IT:Sic2/5:04]	TYLCAxV	TYLCV-IL (A)	406*	1062*	RGBMCST	3.48E-39	2004	1971	2001	1988	W Med	1	W Med	0.51	W Med	1	W Med	0.55
7	TYLCV-Ker -Bou -Mld -Gez	Unknown	TYLCV-like ancestor	2002	2597	RGBMCST	6.34E-44	1938	1794	1973	1964	W Med	0.47	Iran	0.92	ND*	ND	Israel	0.65
8	TYLCV-Bou[IR:Ge29:06]	CLCuGV	TYLCV-Ker (B)	2382	398*	RGBMCSt	1.10E-15	2006	1978	2006	1993	Iran	1	Iran	0.98	Iran	1	Iran	1
9	TYLCV-IL	Unknown	TYLCV-Mld	2058	2343	RGBMCST	5.50E-34	1999	1900	1999	1964	Iran	0.69	Iran	0.85	ND	ND	Israel	0.65
10	TYLCV-IL (A & B)	Unknown	TYLCV-Mld	2541	414	RGBMS	1.20E-10	1999	1900	1999	1964	Iran	0.69	Iran	0.85	ND	ND	Israel	0.65
11	TYLCV-IL	Unknown	TYLCV-Mld	2422	2513	RGBMST	6.94E-07	1999	1900	1999	1964	Iran	0.69	Iran	0.85	ND	ND	Israel	0.65
12	TYLCV-Ker B	Unknown	TYLCV-Ker (A)	1531	1579	RGST	2.04E-08	2006	2001	2006	2003	Iran	1	Iran	0.98	Iran	1	Iran	1
13	TYLCV-OM -Ker (B) -Bou	Unknown	TYLCV-Ker (A)	2145	2335*	RGBm	3.04E-07	1978	1945	1993	1990	Iran	0.99	Iran	0.95	Iran	1	Iran	1
14	TYLCV-Mld	Unknown	TYLCV-Ker	1357	1469	RBMCS	3.85E-10	1938	1862	1973	1964	W Med	0.47	W Med	0.48	ND	ND	Israel	0.65
15	TYLCV-Ker (B) -Bou	ToLCKV	TYLCV-IL	2058	2144*	RB	2.63E-04	1978	1945	1993	1990	Iran	0.99	Iran	0.95	Iran	1	Iran	1
16	TYLCV-IL (C)	Unknown	TYLCV-IL (A & B)	2098	426	RGBMCST	7.56E-18	1985	1900	1995	1988	Iran	1	Iran	0.85	Iran	0.98	Israel	0.6
17	TYLCV-Mld[LB:LBA44:05]	TYLCV-IL (A)	TYLCV-Mld (C)	2496	975	mS	8.72E-04	2005	1990	2005	1997	Israel	1	Israel	0.97	Israel	1	Israel	0.99
18	TYLCV-OM	TYLCV-Ker	TYLCV-IR	1126	1704	BMSt	1.12E-10	1993	1981	1995	1988	Iran	1	Iran	1	Iran	1	Iran	1

1 Positions in the recombinat sequence that are TYLCV-Like.

*Values could not be determined because groups of recombinants were not monophyletic.

It is noteworthy that five of the seven described TYLCV strains are found in Iran. This is a greater number than have been found in any other country (the next highest is two) - a fact which marks Iran as probably being close to the global center of TYLCV diversity.

### TYLCVs display complex inter- and intra-species recombination patterns

As recombination is a major process influencing the evolution of TYLCV and other begomoviruses we analysed 75 TYLCV full length DNA-A-like sequences together with 658 DNA-A and DNA-A-like sequences belonging to other begomoviruses for evidence of (1) TYLCV sequence fragments being transferred into the genomic backgrounds of other species (i.e. events with TYLCV donors) and (2) the genomic fragments of other species being transferred into mostly TYLCV-like genomic backgrounds (i.e. events with TYLCV recipients).

Of the 18 detected recombination events involving TYLCV isolates, 16 were inter-species sequence exchanges (events 1 to 16 in Table 1 and Figure 1) and two were intra-species exchanges (events 17 and 18 in Table 1 and Figure 1). Only four of the 16 inter-species recombination events involved TYLCVs as donors. The recipient species in these four recombination events were western Mediterranean TYLCSVs (events 2, 4 and 5 in Table 1 and Figure 1) and TYLCAxV (event 6 in Table 1) isolates. As has been found previously, two of these events (2 and 4 in Table 1), both involving TYLCSV as a recipient and TYLCV as a donor, were pivotal in the creation of the TYLCAxV and TYLCMalV species [51], [52]. In fact, all three of the TYLCAxV isolates examined (accession numbers AY227892, EU734831 and EU734832), appear to be independently generated convergent recombinants of TYLCSV and TYLCV-IL, highlighting the possibility that, in the Western Mediterranean at least, such recombinants have a high degree of fitness.

The remaining 12 inter-species recombination events involved TYLCVs as recipients of <1000 nucleotide fragments mostly derived from the *rep* genes of either currently undescribed begomovirus species, or species previously detected only in the Middle East and/or India and Asia.

The fact that unique recombination events are detectable within the *rep* sequences of every TYLCV isolate presents somewhat of a problem when it comes to disentangling the evolutionary origins of the various recombinationally derived fragments within this gene. Specifically, without a provably non-recombinant TYLCV *rep* gene in hand it is not possible to objectively judge the accuracy of the parental sequence and recombinant designations given in Table 1 and Figure 1. Put another way, it is possible, if not probable, that some of the parental TYLCV sequences listed in Table 1 are misidentified recombinant sequences and some of the recombinant sequences are misidentified parental sequences.

In this regard, parental and recombinant sequence designations for events 7, 9, and 11 listed in Table 1 were particularly difficult to interpret. Evidence of these recombination events is found within quite divergent TYLCV lineages implying that they either (1) predate the divergence of these lineages or (2) that they are more recent but that the recombinant fragments characterising the events have been propagated by secondary intra-species recombination between the various TYLCV lineages. For example, both the fact that event 7 is found within the TYLCV-Ker, -Mld, -Gez, and -Bou lineages and the evidence of it being overprinted by subsequent recombination events such as 14 in the -Mld lineage, 8 in the –Gez lineage, and 12 in the –Ker(B) lineage, implies that it is a reasonably old recombination event.

With events 9 and 11 on the other hand, it is plausible that a secondary recombination event carrying a fragment baring traces of both events has been transferred from a TYLCV-IL (A) variant into the TYLCV-IL (C) variant (Figure 1). The young age of events 9 and 11 in some of the TYLCV-IL (A) isolates is also implied by how closely some of these isolates resemble TYLCV-Mld (A) isolates within the portion of their genomes upstream of the event 9 5′-breakpoint. For example, over a stretch of 1640 nucleotides the TYLCV-Mld[ES:Alm:99] isolate, and the TYLCV-IL[ES:Alm:Pep:99] isolate, differ at only two nucleotide positions – implying a very young age for the recombination event in *rep* that differentiates them. However, over this 1640 nucleotide fragment these two isolates are also much more closely related to one another than either is to any other TYLCV-Mld or TYLCV-IL isolates. This strongly suggests that after the original inter-species recombination event(s) that resulted in the differentiation of TYLCV-IL from TYLCV-Mld [7], the TYLCV-IL fragment containing traces of events 9, 11 and 10 has, at least once, been transferred back into a TYLCV-Mld isolate (in this case, one very closely resembling TYLCV-Mld[ES:Alm:99]). In recombination analyses such as those which we performed, the resulting recombinants would be virtually indistinguishable from other TYLCV-IL isolates and no recombination would therefore be inferred.

**Figure 2:**
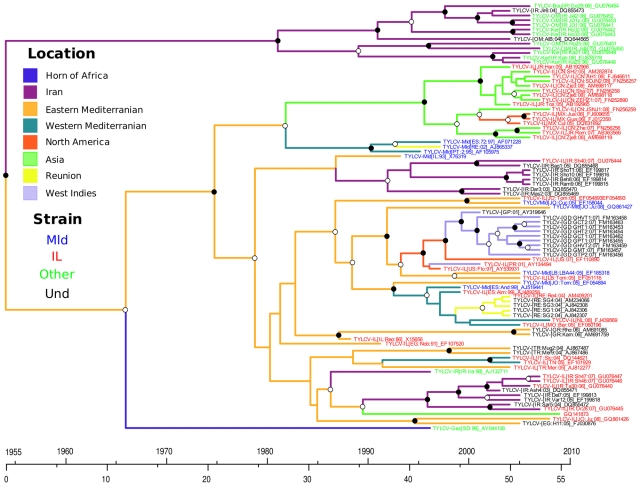
Maximum clade credibility trees constructed from the TYLCV coat protein (CP) dataset. Branches are coloured according to the most probable location state of their descendant nodes. The time-scale of evolutionary changes represented in the tree is indicated by the scale bar below it. Sequence accession numbers are coloured based on the TYLCV strains the sequences belong to. Sequences that are IL and Mld- like but which could not be confidently assigned to either strain because no corresponding full length sequences are available are coloured in black. Whereas filled circles associated with branches indicate >95% posterior probability support, open circles indicate branches with >50% posterior support. Branches with <50% support are unlabeled.

The phylogenetic influences of such undetected cyclical recombination events – where parental viruses are recombinants and recombinants converge on parental viruses – are quite clearly depicted in the MCC tree of TYLCV CP sequences presented in Figure 2. In this tree where the names of IL and Mld isolates are respectively coloured in red and blue, it is immediately obvious that, from the perspective of their CP sequences at least, isolates belonging to each of the strains are more closely related to isolates of the other strain than they are to some isolates of their own strain. This makes it very difficult to phylogenetically determine when recombination events such as those which generated TYLCV-IL from TYLCV-Mld occurred.

### TYLCV probably originated in the Middle East during the first half of the 20^th^ century

Given that recombination is known to confound molecular clock analyses [48], [49] we assembled a mostly recombination-free TYLCV coat protein gene dataset (called the CP dataset). We analysed both this and the full genome (FG) TYLCV datasets with BEAST to determine the time and place where TYLCV originated. While the FG analysis indicated that the mean substitution rates during TYLCV evolution was 4.5×10^−4^ subs/site/year (95% HPD ranging from (2.4×10^−4^ to 6.8×10^−4^), the CP analysis indicated a rate of 7.9×10^−4^ subs/site/year (95% HPD ranging from 4.9×10^−4^ to 1.1×10^−3^). These substitution rate estimates are consistent with the previously published tomato infecting begomovirus full genome substitution rate estimate of 2.44×10^−4^ subs/site/year (95% HPD ranging from 1.3×10^−6^ to 6.1×10^−4^ [47]).

Whereas the age of the most recent common TYLCV ancestor was estimated to be 293 years (95% HPD 138–515) using the FG dataset it was estimated to be only 56 years (95% HPD ranging between 35–80) using the CP dataset. These contradictory date estimates are almost certainly due to every one of the main TYLCV lineages in the FG dataset being different inter-species recombinants with highly divergent *rep* genes (Figure 1). It is expected that with the FG dataset, the much older dates of the last common ancestors of these highly divergent recombinationally acquired *rep* genes would have legitimately pushed the estimated of the most recent TYLCV common ancestor much deeper into the past [47], [53] (i.e. the estimated date is expected to be somewhere between the actual date of the Rep MRCA and the date of the MRCA of the rest of the genome).

Despite the biasing influence of recombination in the FG dataset on the estimated timing of evolutionary events, both the FG and CP analyses clearly indicated that the most recent common ancestor of the TYLCVs probably resided in the Middle East – either somewhere near Iran (posterior state probability, or PSP,  = 0.53 for the FG dataset and 0.15 for the CP dataset, Figure S3) or somewhere in the Eastern Mediterranean (PSP = 0.13 for the FG dataset and 0.48 for the CP dataset, Figure S3). The PSP estimate of 0.53 for the FG dataset means that 53% of similarly plausible phylogenetic trees assessed during the analysis are consistent with this ancestral sequence being resident in Iran. Thus 68% of trees assessed during the FG analysis and 61% assessed during the CP analysis are consistent with the most recent common ancestor of the TYLCVs being resident in the Middle East (i.e. Iran PSP + Eastern Mediterranean PSP). These percentages can be considered probability estimates which, although not higher than 95%, indicate that it is more than three times more probable that the most recent common ancestor of the TYLCVs was located near either Iran or in the Eastern Mediterranean than it is that the ancestor was located in the next most probable region (the Western Mediterranean which has an associated PSP  = 0.085 for the FG dataset and 0.19 for the CP dataset, Figure S3).

Importantly, this pattern was recapitulated even in sets of sub-sampled datasets designed to mitigate potential sampling biases in the complete CP and FG datasets. In all ten of the sub-sampled CP and FG datasets the most probable location of the TYLCV MRCA was either the region around Iran (CP and FG datasets with respective mean PSPs  = 0.26 and 0.25) or the Eastern Mediterranean (CP and FG datasets with respective mean PSPs  = 0.4 and 0.12; Figure S3). Also, when we reran our analyses with the full datasets in such a way that the location state designations of all of the sequences were randomized throughout the MCMC procedure, the maximum PSP achieved at the root node for the most sampled location never exceeded 0.18 for the FG dataset and 0.22 for the CP dataset – both much lower than the maximum root node PSPs obtained without the location state randomisation setting (which were 0.53 and 0.48 for the FG and CP datasets respectively). Together these tests indicated that sampling biases had not obviously influenced our identification of the Middle East as the region where TYLCV most probably originated.

### The Mediterranean basin (and not Iran) is the source of the global TYLCV epidemic

**Figure 3:**
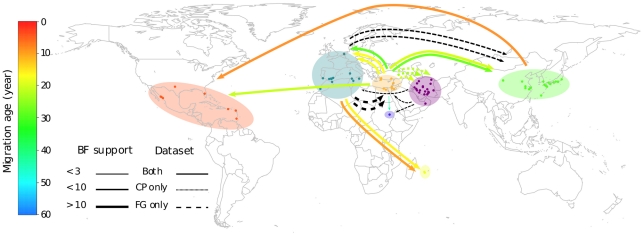
TYLCV migration events inferred using the coat protein (CP) and full genome (FG) datasets. Sampling locations are indicated using circles that are coloured depending on the discreet sequence groupings they were assigned to during our phylogeography analyses (indicated by transparent coloured areas). Virus movements implied by location state transitions along the branches of the CP (see Figure 2) and FG MCC trees are indicated using arrows. Arrow colours depict the mean ages (in years) of the movements that they represent (inferred using the CP dataset and coloured according to the colour scale on the left of the figure). The thickness of arrows indicating movements between two locations indicate the over-all Bayes factor test support for epidemiological linkage between the locations. Whereas individual migration events inferred with both the CP and FG datasets are represented using solid arrows, events inferred with only the CP or the FG dataset are represented with dotted and dashed lines respectively.

To pinpoint the source of the TYLCV variants that are spreading throughout the world, we retraced the movement patterns of TYLCVs over the past 50 years. Figure 2 is a phylogenetic depiction of TYLCV movement patterns (based on the CP dataset MCC tree) in which the tree branches have been coloured based on the most probable locations of their associated virus lineages such that a colour change between two connected nodes implies a probable migration event. In addition, a plausible spatio-temporal animation of TYLCV movements since the time of the most recent TYLCV common ancestor can be visualised by opening in GoogleEarth (http://earth.google.com) the Dataset S1.kml (FG dataset) and Dataset S2.kml (CP dataset). Figure 3 summarises the results presented in these files. It is important to stress that in these analyses, we only considered the nine and seven discreet locations respectively studied in the CP and FG datasets. It must therefore be borne in mind that the locations indicated for ancestral viruses and the movement patterns inferred from these are simply the most plausible given the studied sampling locations – i.e. that actual locations of ancestral sequences and movement pathways may have included locations outside those that we have considered.

Among the locations that we have considered, the FG and CP datasets respectively indicate that the global dispersal of TYLCV has involved at least 15 and 17 discrete migration events. As these viral movements (or geographical location state transitions) were inferred from node states of the FG and CP MCC trees, we only summarise the realisations of a potentially rich history of location state transitioning. The reason for this is that the geographical location states mapped to the various tree nodes reflect the starting and ending points of various movements – they do not recapture the potentially long and winding routes taken during these journeys.

Both the FG and CP datasets indicate that TYLCVs have moved at least twice from the Eastern Mediterranean to Asia (Bayes factors, or BF,  = 11.5 and 3.6 where a BF >100 represents decisive support, a BF >10.0 represents strong support, a BF >3.2 represents substantial support and a BF <3.2 is not well supported [44], three times to the Mediterranean (BF  = 15.7 and 1209) and once to North America (BF  = 2.36 and 11.8). The FG analysis also indicated that two independent TYLCV movements have occurred from the Western Mediterranean to Asia (BF  = 3.6). Consistent with previous reports [54], both the FG and CP datasets also indicate two migration events from the Western Mediterranean to the southern Indian Ocean island of Reunion (BF = 17.6, 729). It is also noteworthy that with the FG dataset two migration events are inferred from the Western Mediterranean to the Eastern Mediterranean (BF = 15.7; although no corresponding migrations were inferred with the CP dataset), indicating that TYLCV movements between these regions may be bidirectional.

Although the FG analysis indicated that TYLCV probably originated near Iran, this analysis indicated only weak support for three early virus movements out of Iran to the Eastern Mediterranean (BF  = 2.64), the Horn of Africa (BF  = 2.01) and the Western Mediterranean (BF  = 0.64). In the CP analysis where the Eastern Mediterranean rather than the Iranian region was identified as the probable origin of TYLCV, three independent, decisively supported (BF  = 265), migration events from the Eastern Mediterranean to Iran were inferred, possibly explaining the broad degree of TYLCV diversity found in the latter region.

Finally, our analysis supports the hypotheses that TYLCV-IL has been independently introduced to the New World, once from the region around the Eastern Mediterranean (BF  = 2.36 and 11.8 for the FG and CP datasets respectively) and once from Asia (BF  = 13.6 and 45.7 for the FG and CP datasets respectively; [16]).

Collectively these data indicate that although the region around Iran is a center of TYLCV diversity and is possibly also the region where the species originated, it has not been the direct source of the TYLCV variants that are currently spreading worldwide. This means that novel pathogenic TYLCV variants that arise in this region will probably be less of a threat to global agriculture than those arising closer to more internationally connected regions such as the Mediterranean basin.

### The geographical and temporal origins of TYLCV recombinants

Our preliminary recombination analysis indicated that all of the detectable recombinants that discernibly contained TYLCV-like sequences had been sampled in the Mediterranean basin and the Middle East. We suspected that within this region there might be geographical recombination hotspots. By mapping the 18 detected TYLCV recombination events to the FG and CP MCC trees determined during our phylogeography analysis, it was possible for us to approximate the locations where and the times when the recombination events most likely occurred. For each recombination event in each tree this involved identification of the nodes representing (1) the last common ancestor of the recombinants (referred to as RecAnc in Table 1) and (2) the last TYLCV ancestor not sharing evidence of the same recombination event (referred to as Non-RecAnc in Table 1). The dates and locations of the sequences at these two nodes in the MCC trees were assumed to bound the date when, and the location where, the recombination event occurred.

Whereas it was possible to use this approach to infer dates and locations for all 18 of the recombination events with the FG dataset, groups of recombinant TYLCV-IL and –Mld sequences sharing evidence of events 7, 9, 10, 11 and 14 were not monophyletic in the CP tree (probably for reasons explained above in the recombination analysis section; Figure 2), meaning that locations and dates could not be properly inferred for these recombination events using the CP dataset. Despite this, the CP dataset yielded much tighter estimates of recombination dates than the FG dataset, possibly due to its being free of the confounding effects of the inter-species recombination events found in the latter.

The FG and CP datasets nevertheless indicated locations where recombination events had occurred that were generally in good agreement with one another (compare orange and blue bars in Figure S4) and recombination date estimates that had broadly overlapping 95% HPDs (compare orange and blue bars in Figure S5). The exceptions were the five “problematic” events (7, 9, 10, 11 and 14) mentioned previously. For these the FG and CP datasets yielded support for recombination events having occurred in different locations. For example with events 9, 10 and 11 the FG dataset indicated that the RecAnc and Non-RecAnc sequences most probably resided near Iran, the CP dataset indicated that the Non-RecAnc sequence most probably resided near the Eastern Mediterranean (with the location of the RecAnc sequence remaining undetermined for the CP dataset).

Nevertheless, the clear pattern emerging from these analyses was that all 18 of the detected TYLCV recombination events occurred either in the Western Mediterranean, the Eastern Mediterranean or near Iran. Collectively these geographical locations (representing 58% of the sequence) accounted for more than 80% of the posterior probability distribution for every ancestral sequence used to infer the locations of every recombination event. Based on dates inferred from the CP MCC tree, these recombination events were also mostly all quite recent with the oldest (events 7 and 14) having most probably occurred some time after 1964 (Table 1 and Figure S5). If one discounts the “problematic” recombination events 7, 9, 10, 11 and 14, the remaining thirteen events have all most probably occurred since 1985.

Nine of these thirteen events most probably occurred near Iran or Israel with both the FG and CP analyses indicating that Iran was the most probable site of eight of them (supported for all events other than events 1 and 16 by the location state probabilities of all the relevant RecAnc and Non-RecAnc sequences in both the FG and CP datasets). Besides being the most probable origin of TYLCV and the center of TYLCV diversity, the Middle East in general, and Iran in particular, is therefore also apparently the region where most of this virus' evolutionary change through recombination has occurred.

In this regard it is interesting that recombination events 2, 4, 5 and 6, the only events that almost certainly occurred outside the Middle East, are also the only four involving TYLCV sequences as donors (i.e. such that a minority of the recombinant's genome consists of TYLCV-like sequences). Although this difference between the character of TYLCV recombination events occurring inside and outside the Middle East may be coincidental, it could also be indicative of an important evolutionary trend associated with the migration of viruses into environments different from those in which they evolved.

The observed pattern is in fact what one might expect to occur with recombining invasive virus species. For example, it is expected that viruses residing in the locations where they evolved would be well adapted to seasonal changes in the mix of host and vector genotypes that typify their home environments. One might expect both that these adaptations would provide them with a “home environment advantage” over invasive newcomers and that the genetic underpinnings of these adaptations would be distributed throughout their genomes. The invasive newcomers, however, would not be invasive unless they had some specific, especially adaptive genetic trait that provided them with their invasive phenotype. When such indigenous and invasive viruses recombine, the fittest of their offspring would probably be those that incorporate the invader's highly adaptive traits within an indigenous genetic background. Unless TYLCV and TYLCSV only replicate within genetically homogeneous cultivated tomato species and are epidemiologically unaffected by local variations in host species distributions across the Mediterranean and Middle East, it is conceivable that both have an advantage in their respective home environments. The net result may be that in the Middle East when TYLCVs recombine with viruses originating in India or Africa the TYLCVs are the better acceptors whereas in the Western Mediterranean they are better as donors to indigenous viruses like TYLCSV.

### A plausible history of TYLCV

To retrace the global movements of TYLCV we considered phylogeographic inferences made with both the FG and CP datasets. However, given that the estimated calendar dates of movement events differed between the FG and CP analyses and the probable impact that inter-species recombination has had on evolution rates estimated with the FG dataset, we used results obtained with the mostly recombination-free CP dataset to estimate the timing of key events during the evolution and dissemination of TYLCV (summarised in Figure 3). It is important to reiterate here that both the age, location and migration route estimates that follow are associated with degrees of uncertainty and that the descriptive history we provide is simply the most plausible given an admittedly sparse TYLCV sequence dataset and imperfect analytical tools. Nevertheless, although the Bayesian analyses underlying the description do not account for important factors such as natural selection, they do provide us with 95% HPD intervals that are an honest expression of the uncertainty surrounding the various date, location and migration route estimates that we infer.

At some point between 1937 and 1952 (95% HPD 1905–1972), a virus arose somewhere within the Middle East which was the first recognisable TYLCV. By ∼1952 this “first” TYLCV lineage had evolved into the most recent common ancestor of all known contemporary TYLCVs. It is possible, although in no way certain, that this virus was a recombinant that had inherited the majority of its genome from an earlier TYLCV prototype but a large portion of its *rep* gene and its origin of virion strand replication from some other unknown (but possibly Asian) begomovirus species (see event 7 in Figure 1, Table 1 and Figures S4 and S5). It is also plausible that the immediate descendants of this virus were responsible for the Middle Eastern TYLCD epidemics of the early 1960s [4], [5].

There is good evidence from our analysis that during the 1960s these viruses evolved within the Middle East to yield prototypical versions of the TYLCV-Gez strain in the Eastern Mediterranean (PSP  = 0.65), by ∼1964 (95% HPDs 1948–1978), the TYLCV-Mld strain also in the Eastern Mediterranean (PSP  = 0.90) by ∼1973 (95% HPDs 1963–1982) and the TYLCV-Ker strain in the region of Iran (PSP  = 0.97) by ∼1979 (95% HPDs 1964–1992). Later, between 1993 and 2006 (95% HPD 1986–2006) and also probably in Iran (PSP  = 0.98–1.00), a recombination event between a TYLCV-Ker variant and CLCuGV (event 8 in Table 1 and Figure 1) created the first member of TYLCV-Bou, the most recently evolved of the seven currently described TYLCV strains.

Although both inter- and intra-species recombination events involving early TYLCV variants probably persistently occurred within the broader Middle East during these years, the first of these that would come to largely differentiate the seven current TYLCV strains probably occurred somewhere in this region (PSP  = 0.80–0.76) between 1964 and the mid 1970s (95% HPD 1948–1999). This event (or possibly a series of events), traces of which are possibly evident in events 9, 10 and 11 in Table 1 and Figure 1, yielded the founder of the IL strain. Similar recombination events between either TYLCV-Mld or -IL (it is unclear which) and TolCRV somewhere in the Middle east (PSP  = 0.97–1.0) between 1985 and 1996 (95% HPDs 1978– 1996; event 1 in Table 1 and Figure 1) and between TYLCV-Mld or -IL and ToLCKV near Iran (PSP  = 0.99–1.0) between 1996 and 2000 (95% HPDs 1991–2003; event 3 in Table 1 and Figure 1) would respectively yield the first members of what are currently known as the TYLCV-IR and -OM strains.

At some point between 1981 and 1989 (95% HPDs 1971–1993) the world-wide dissemination of TYLCV began when a TYLCV-IL virus (most likely from the Eastern Mediterranean), moved to, and became established within, the Western Mediterranean. This trip was later repeated at least once by a TYLCV-Mld virus between 1990 and 2001 (95% HPDs 1982–2003). Although the polarity of the movement is uncertain (the FG and CP datasets conflict on this point), additional movements of IL viruses between the Middle East and the Western Mediterranean also occurred during this period. Viruses within the newly established Western Mediterranean TYLCV-Mld and -IL populations were then transported to Asia between 1989 and 1996 (95% HPDs 1983–1996) and the Indian Ocean island of Reunion between 1991 and 2002 (95% HPDs 1987–2003).

At least two other long distance movements of IL viruses to Asia also occurred from the Middle East between 1981 and 1999 (95% HPD 1970–1999). Whereas the trans-Atlantic movement of a Middle Eastern TYLCV-IL virus to the New World probably happened between 1992 and 1994 (95% HPD 1988–1994) – within two years of the first TYLCVs being sampled there [15] – the trans-Pacific transport of an Asian TYLCV-IL virus (a descendant of one of the lineages introduced from the Middle East) to North America probably only occurred between 1999 and 2003 (95% HPD 1996–2004).

### Concluding remarks

We have described how within thirty years of their Middle Eastern origin, both TYLCV-Mld and the TYLCV-IL lineage have ascended to the point where they are today ranked among the greatest biotic threats to tomato production world-wide [55]. This emergence has been so swift that no precise estimates of either their current or projected future economic impacts exist. The epidemiological, evolutionary and ecological impacts of their movements are probably even harder to predict although in this regard patterns seen in the Western Mediterranean where they have spent their greatest time outside the Middle East will possibly prove informative [56]–[58]. For example, given the high frequencies of inter-species TYLCV recombination events that we have mapped to the Middle East, it is perhaps reasonable to expect that, as has happened in the Western Mediterranean [7], [22], [51], TYLCVs introduced to the Americas, the southern Indian Ocean, and Asia will recombine with viruses indigenous to these regions. While it is impossible to predict how evolutionarily productive any such recombination events will be, the possibility remains that TYLCV genetic material within the context of mostly indigenous recombinant begomovirus genomes could shortly begin showing up in Asia, the Indian Ocean islands and the Americas. We envision that tracking the movements of the various TYLCV invasion fronts and monitoring virus sequence data before the fronts hit and in the years thereafter could prove very fruitful in our endeavours to answer some key questions relating to the economic, epidemiological, ecological and evolutionary impacts of such plant virus invasions.

## Supporting Information

Figure S1 Maximum likelihood phylogenetic tree (with GTR + G_4_ selected as the best fit model by RDP3) and pair-wise sequence similarity matrix of 75 virus isolates representing the seven different TYLCV strains (denoted by different colours on the tree branches). The phylogeny is rooted using TYLCMLV. The colours in the matrix represent the pair-wise similarities indicated on the colour scale. Similarity scores beneath the accepted begomovirus species demarcation cut-off, 89% are in a yellow scale, scores in the strain range, between 89% to 93% are in a light blue scale and scores in the variant range between 93% and 100% are in a blue scale.
(1.93 MB TIF)

Figure S2 Sampling locations of Iranian TYLCV isolates. Small circles at sample sites are coloured depending on whether (green) or not (black) TYLCVs were cloned from samples collected at the sites. Sites where TYLCVs were sampled in other studies are given in blue. Coloured areas represent the known geographical distributions within Iran of the different TYLCV strains.
(0.59 MB TIF)

Figure S3 The posterior probability distribution indicating the most probable geographical locations of the last common TYLCV ancestor. Bars indicate Bayesian posterior probabilities that the last common TYLCV ancestor resided in the various sampling locations. Blue bars represent inferences of ancestral sequence locations made using the full genome (FG) dataset and orange bars represent those made using the coat protein (CP) dataset.
(0.11 MB TIF)

Figure S4 The approximate geographical origins of TYLCV recombinants. Bars indicate Bayesian posterior probabilities that sequences closely related to the ancestral recombinant sequence (the last non-recombinant most recent common ancestor or the recombinants and the most recent common recombinant ancestor of the recombinant(s)) resided in the various sampling locations. Blue bars represent inferences of ancestral sequence locations made using the full genome dataset and orange bars represent those made using the coat protein (CP) dataset. Whereas the darker bars indicate the probability that the last non-recombinant ancestor of the recombinant sequences was situated in the specified locations, the lighter bars indicate the probability that the last common ancestor of all sampled recombinants was located in the regions. In cases where only one recombinant has been sampled the probability associated with the location where the recombinant was sampled is 1. Wherever it was not possible to directly infer the location of the last non-recombinant ancestor (see M&M for details on how locations were estimated) estimates are marked with an asterisk.
(0.68 MB TIF)

Figure S5 Dating of TYLCV recombination events. The upper and lower bounds of the coloured bars respectively indicate the most probable range of dates when the various recombination events might have occurred. The thinner error bars indicate the upper and lower 95% HPD intervals of the date estimates. Refer to the M&M to see how the dates were calculated.
(1.04 MB TIF)

Table S1 Dataset details.
(0.07 MB PDF)

Dataset S1 Google earth file with an animation of TYLCV migration inferred using the full genome dataset.
(0.07 MB ZIP)

Dataset S2 Google earth file with an animation of TYLCV migration inferred using the CP dataset.
(0.06 MB ZIP)
