# Supplementary figures and images for: The Spread of Tomato Yellow Leaf Curl Virus from the Middle East to the World
Source: PLoS Pathog. 2010 Oct 28;6(10):e1001164. doi: 10.1371/journal.ppat.1001164 (PMC2965765; doi:10.1371/journal.ppat.1001164)

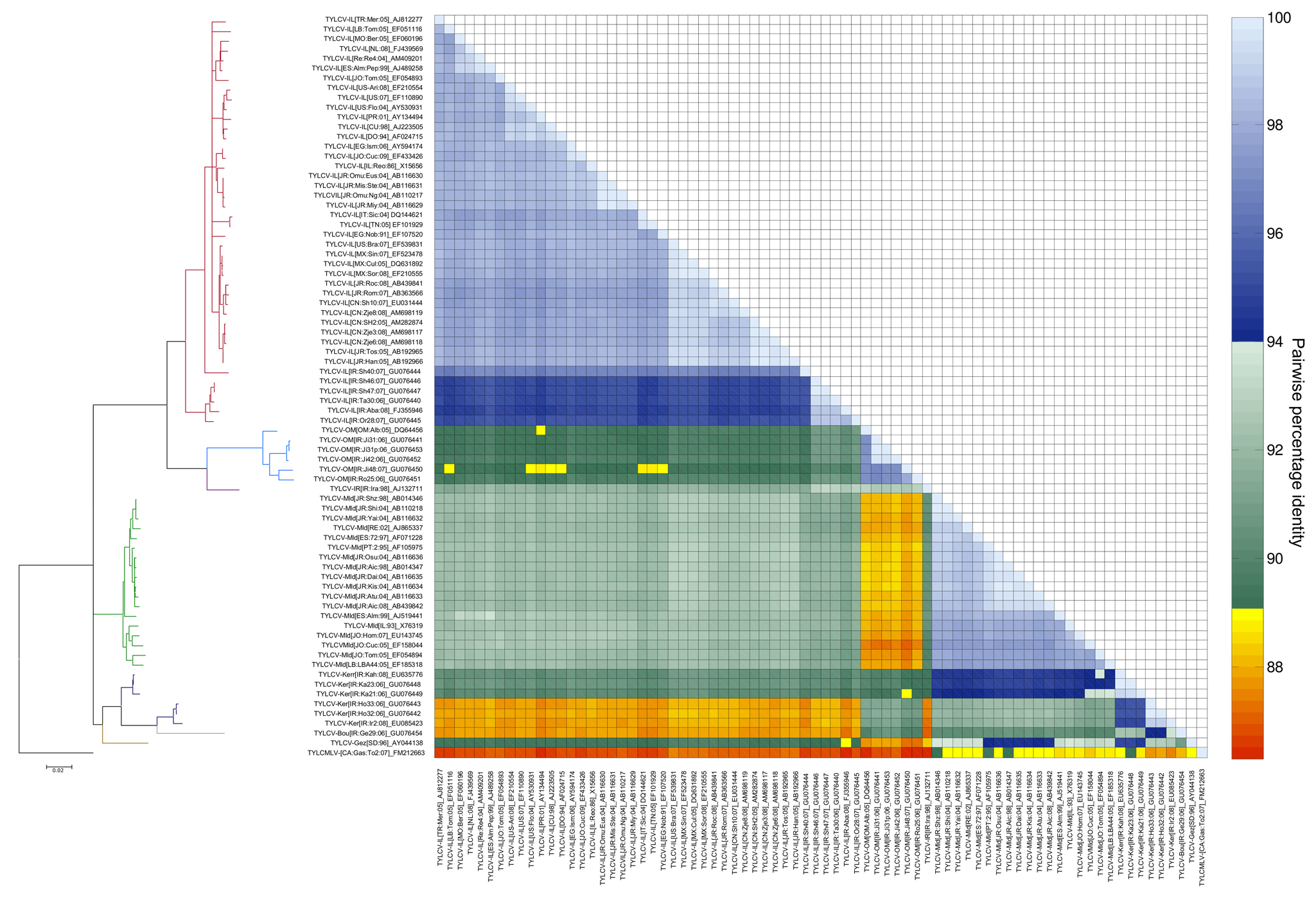

Supplement: Figure S1 — Maximum likelihood phylogenetic tree (with GTR + G4 selected as the best fit model by RDP3) and pair-wise sequence similarity matrix of 75 virus isolates representing the seven different TYLCV strains (denoted by different colours on the tree branches). The phylogeny is rooted using TYLCMLV. The colours in the matrix represent the pair-wise similarities indicated on the colour scale. Similarity scores beneath the accepted begomovirus species demarcation cut-off, 89% are in a yellow scale, scores in the strain range, between 89% to 93% are in a light blue scale and scores in the variant range between 93% and 100% are in a blue scale. (1.93 MB TIF) [file ppat.1001164.s001.tif]

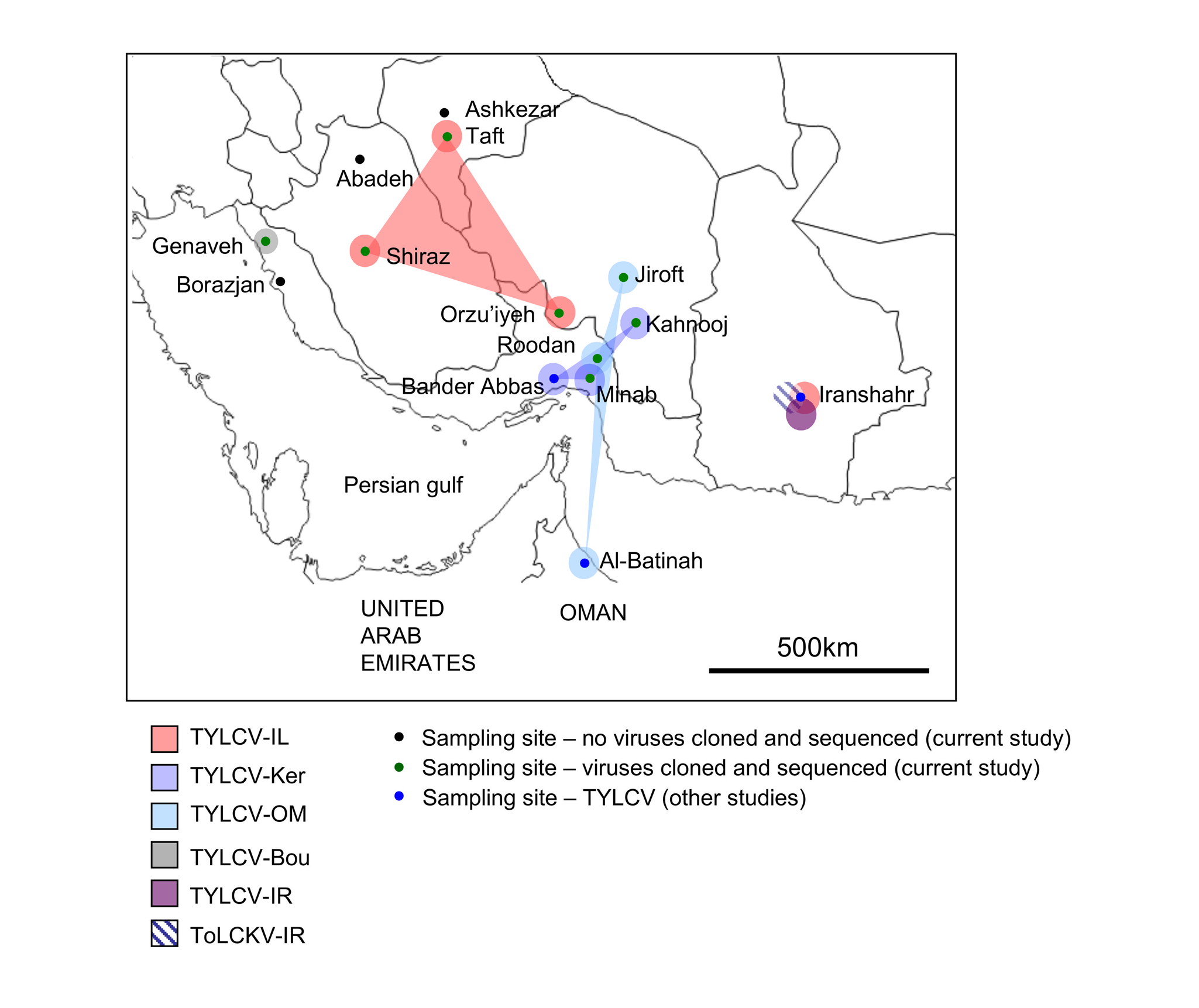

Supplement: Figure S2 — Sampling locations of Iranian TYLCV isolates. Small circles at sample sites are coloured depending on whether (green) or not (black) TYLCVs were cloned from samples collected at the sites. Sites where TYLCVs were sampled in other studies are given in blue. Coloured areas represent the known geographical distributions within Iran of the different TYLCV strains. (0.59 MB TIF) [file ppat.1001164.s002.tif]

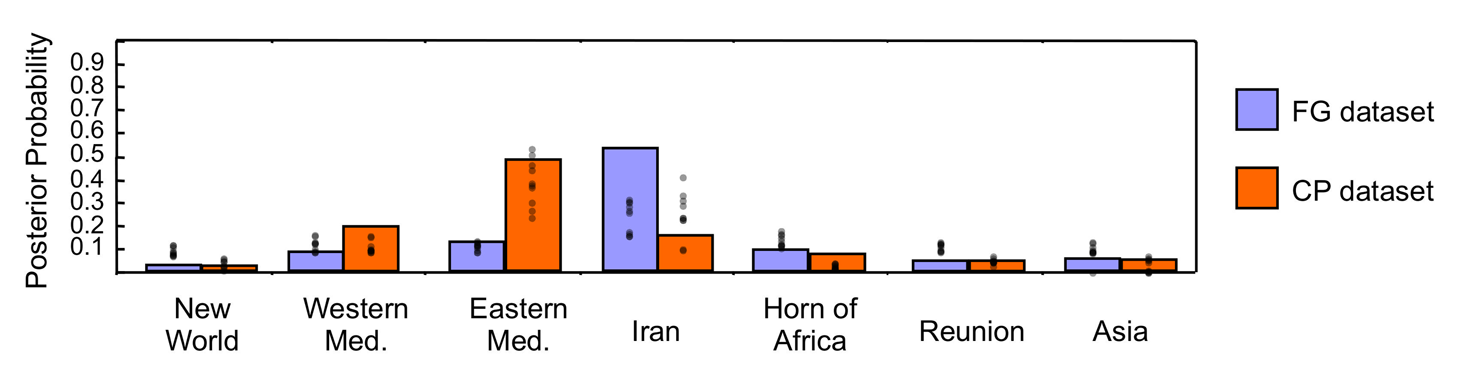

Supplement: Figure S3 — The posterior probability distribution indicating the most probable geographical locations of the last common TYLCV ancestor. Bars indicate Bayesian posterior probabilities that the last common TYLCV ancestor resided in the various sampling locations. Blue bars represent inferences of ancestral sequence locations made using the full genome (FG) dataset and orange bars represent those made using the coat protein (CP) dataset. (0.11 MB TIF) [file ppat.1001164.s003.tif]

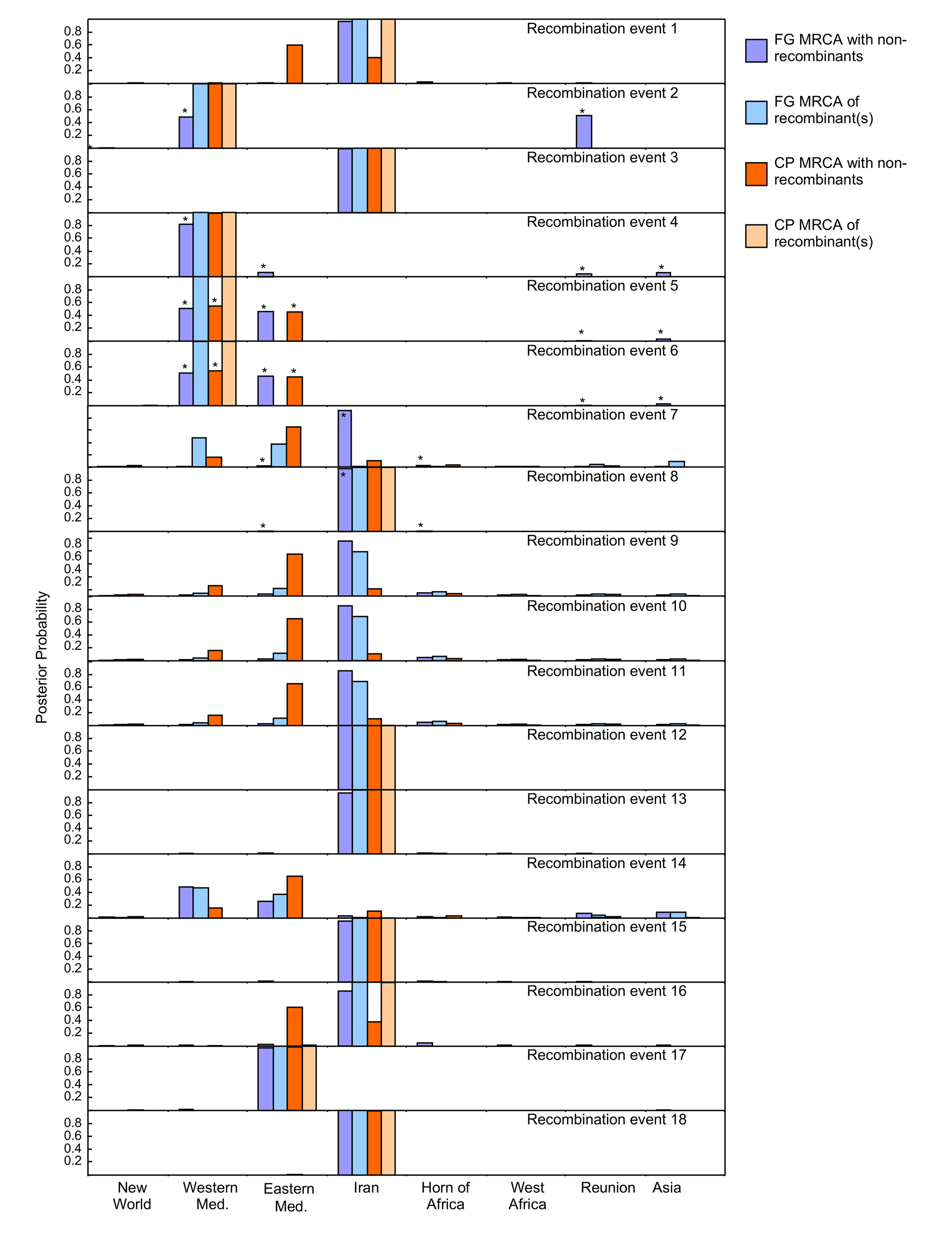

Supplement: Figure S4 — The approximate geographical origins of TYLCV recombinants. Bars indicate Bayesian posterior probabilities that sequences closely related to the ancestral recombinant sequence (the last non-recombinant most recent common ancestor or the recombinants and the most recent common recombinant ancestor of the recombinant(s)) resided in the various sampling locations. Blue bars represent inferences of ancestral sequence locations made using the full genome dataset and orange bars represent those made using the coat protein (CP) dataset. Whereas the darker bars indicate the probability that the last non-recombinant ancestor of the recombinant sequences was situated in the specified locations, the lighter bars indicate the probability that the last common ancestor of all sampled recombinants was located in the regions. In cases where only one recombinant has been sampled the probability associated with the location where the recombinant was sampled is 1. Wherever it was not possible to directly infer the location of the last non-recombinant ancestor (see M&M for details on how locations were estimated) estimates are marked with an asterisk. (0.68 MB TIF) [file ppat.1001164.s004.tif]

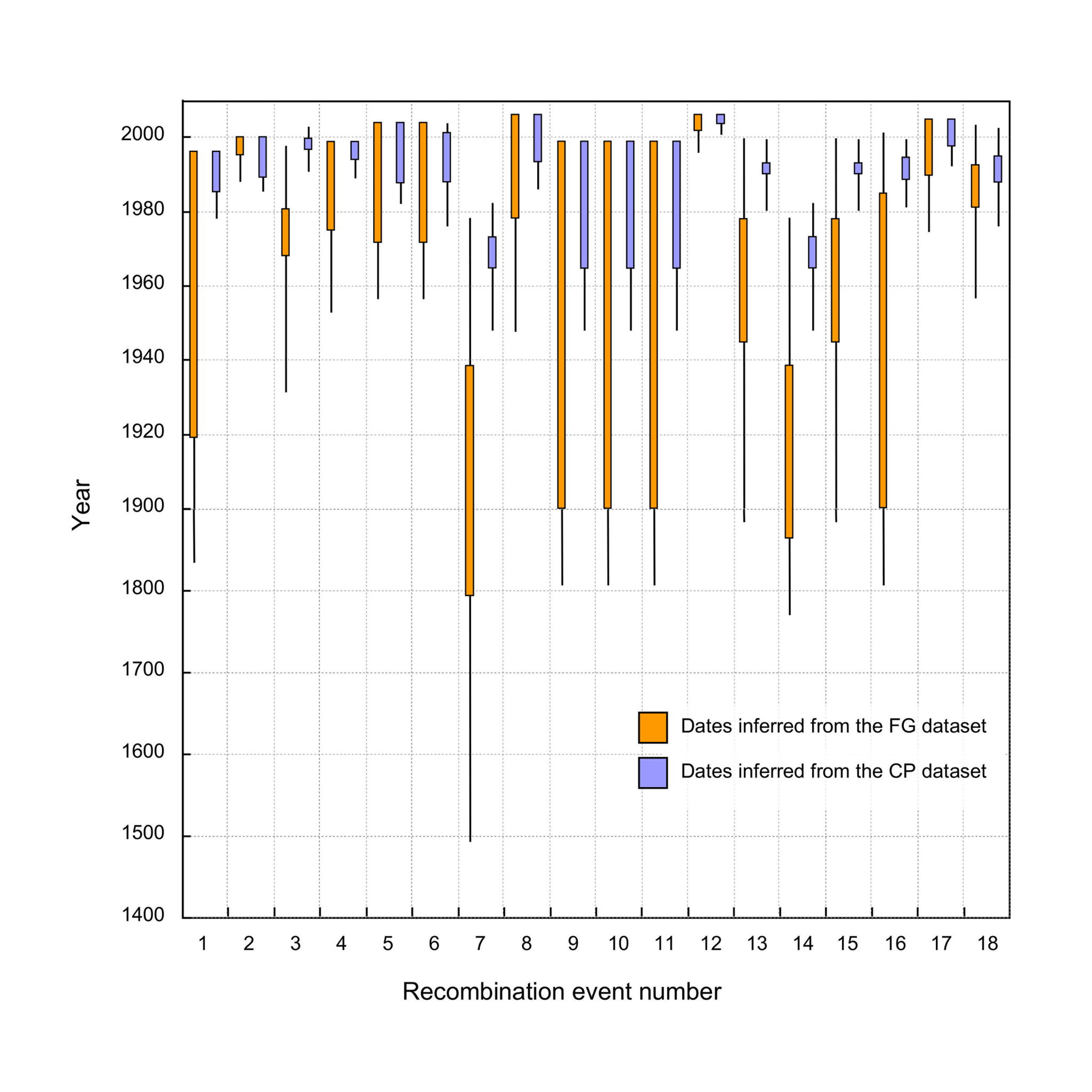

Supplement: Figure S5 — Dating of TYLCV recombination events. The upper and lower bounds of the coloured bars respectively indicate the most probable range of dates when the various recombination events might have occurred. The thinner error bars indicate the upper and lower 95% HPD intervals of the date estimates. Refer to the M&M to see how the dates were calculated. (1.04 MB TIF) [file ppat.1001164.s005.tif]
