# Supplementary material for: The Spread of Tomato Yellow Leaf Curl Virus from the Middle East to the World
Source: PLoS Pathog. 2010 Oct 28;6(10):e1001164. doi: 10.1371/journal.ppat.1001164 (PMC2965765; doi:10.1371/journal.ppat.1001164)
Supplement: Table S1 — Dataset details. (0.07 MB PDF) [file ppat.1001164.s006.pdf]

Table S1: Dataset details

Accession Number	Collection year	Country /Region	Dataset	Longitude	Lattitude	Groupe	Centroid logitude	Centroid lattitude
AY044138	1996	Sudan	FG	33.5	14.5	Horn of Africa	11.45	36.41
AB116631	2000	Japan	FG	130.47	32.61	Asia	32.90	127.01
AB192966	2004	Japan	FG	133.4	33.5	Asia	32.90	127.01
AB192965	2004	Japan	FG	133.4	33.4	Asia	32.90	127.01
AB116636	2002	Japan	FG	135.5	34.69	Asia	32.90	127.01
AB116635	2002	Japan	FG	135.62	34.71	Asia	32.90	127.01
AB116634	2002	Japan	FG	140.62	36.38	Asia	32.90	127.01
AB116633	2002	Japan	FG	132.45	34.4	Asia	32.90	127.01
AB116632	2002	Japan	FG	138.32	34.87	Asia	32.90	127.01
AB116629	2001	Japan	FG	131.42	31.91	Asia	32.90	127.01
AB014347	1996	Japan	FG	136.91	35.18	Asia	32.90	127.01
AB014346	1996	Japan	FG	138.38	34.98	Asia	32.90	127.01
AB116630	1999	Japan	FG	129.96	32.91	Asia	32.90	127.01
AB363566	2007	Japan	FG	127.82	26.37	Asia	32.90	127.01
AM282874	2006	China/Shanghai	FG	121.48	31.22	Asia	32.90	127.01
AM698117	2006	China/Zhejiang	FG	118	31	Asia	32.90	127.01
AM698118	2006	China/Zhejiang	FG	118	31	Asia	32.90	127.01
AM698119	2006	China/Zhejiang	FG	118	31	Asia	32.90	127.01
FJ646611	2008	China	FG	104.2	35.86	Asia	32.90	127.01
FN252890	2007	China/Zhejiang	FG	120.2	30.3	Asia	32.90	127.01
FN256256	2007	China/Zhejiang	FG	120.2	30.3	Asia	32.90	127.01
FN256257	2008	China/Shandong	FG	116	35	Asia	32.90	127.01
FN256258	2007	China/Shanghai	FG	121	31	Asia	32.90	127.01
FN256259	2008	China/Jiangsu	FG	118	32	Asia	32.90	127.01
GQ141873	2009	South Korea	FG	129.08	35.18	Asia	32.90	127.01
DQ845787	2005	Israel	FG	34.8	31.9	E Mediterranean	31.75	34.93
EF051116	2005	Lebanon	FG	35.86	33.85	E Mediterranean	31.75	34.93
EF054893	2005	Jordan	FG	36.24	30.59	E Mediterranean	31.75	34.93
EF054894	2005	Jordan	FG	36.24	30.59	E Mediterranean	31.75	34.93
EF107520	1991	Egypt	FG	29.78	30.78	E Mediterranean	31.75	34.93
AY594174	2000	Egypt	FG	32.26	30.59	E Mediterranean	31.75	34.93
EF158044	2005	Jordan	FG	36.24	30.59	E Mediterranean	31.75	34.93
GQ861426	2008	Jordan	FG	36.24	30.59	E Mediterranean	31.75	34.93
GQ861427	2008	Jordan	FG	36.24	30.59	E Mediterranean	31.75	34.93
EF185318	2005	Lebanon	FG	35.86	33.85	E Mediterranean	31.75	34.93
X15656	1988	Israel	FG	34.85	31.05	E Mediterranean	31.75	34.93
X76319	1993	Israel	FG	34.85	31.05	E Mediterranean	31.75	34.93
AJ812277	2004	Turkey	FG	34.63	36.8	E Mediterranean	31.76	34.94
AF071228	1997	Spain	FG	-2.69	36.75	W Mediterranean	36.29	1.29
AF105975	1995	Portugal	FG	-7.93	37.02	W Mediterranean	36.29	1.29
AJ489258	1999	Spain	FG	-2.81	36.78	W Mediterranean	36.29	1.29
AJ519441	1999	Spain	FG	-2.81	36.78	W Mediterranean	36.29	1.29
AY227892	2000	Spain	FG	-4.05	36.75	W Mediterranean	36.29	1.29
AY702650	2002	Morocco	FG	-9.6	30.41	W Mediterranean	36.29	1.29
AY736854	2002	Tunisia	FG	10.99	35.62	W Mediterranean	36.29	1.29
DQ144621	2004	Italy/Sicily	FG	14.73	36.93	W Mediterranean	36.29	1.29
EF060196	2005	Morocco	FG	-2.33	34.92	W Mediterranean	36.29	1.29
EF101929	2005	Tunisia	FG	10.18	36.78	W Mediterranean	36.29	1.29
FJ439569	2008	Netherlands	FG	5.29	52.13	W Mediterranean	36.29	1.29
L27708	1992	Spain	FG	-2.47	36.84	W Mediterranean	36.29	1.29
X61153	1998	Italy/Sardinia	FG	9.01	40.12	W Mediterranean	36.29	1.29
AF271234	1999	Spain	FG	-2.61	36.76	W Mediterranean	36.29	1.29
Z25751	1992	Spain	FG	-1.58	37.4	W Mediterranean	36.29	1.29
Z28390	1991	Italy/Sicily	FG	14,66	37,4	W Mediterranean	36.29	1.29
AJ132711	1996	Iran	FG	60.86	29.51	Iran	28.60	56.04
DQ644565	2004	Oman	FG	55.92	21.51	Iran	28.60	56.04
EU635776	2005	Iran	FG	57.82	28.27	Iran	28.60	56.04
GU076449	2006	Iran	FG	57.02	30.23	Iran	28.60	56.04
GU076448	2006	Iran	FG	57.08	30.25	Iran	28.60	56.04
GU076451	2006	Iran	FG	57.18	27.57	Iran	28.60	56.04
GU076445	2007	Iran	FG	55.68	28.92	Iran	28.60	56.04
GU076454	2006	Iran	FG	50.58	29.58	Iran	28.60	56.04
GU076440	2006	Iran	FG	54.18	31.73	Iran	28.60	56.04
GU076453	2006	Iran	FG	57.73	28.64	Iran	28.60	56.04
GU076441	2006	Iran	FG	57.71	28.58	Iran	28.60	56.04
GU076442	2006	Iran	FG	57.05	27.06	Iran	28.60	56.04
GU076443	2006	Iran	FG	57.05	27.1	Iran	28.60	56.04
GU076444	2007	Iran	FG	52.53	29.58	Iran	28.60	56.04
GU076452	2006	Iran	FG	57.72	28.64	Iran	28.60	56.04
GU076446	2007	Iran	FG	52.49	29.59	Iran	28.60	56.04
GU076447	2007	Iran	FG	52.52	29.57	Iran	28.60	56.04
GU076450	2007	Iran	FG	57.72	28.62	Iran	28.60	56.04

AY134494	2001	Puerto Rico	FG	-66.93	17.97	North America	24.24	-90.12
AY530931	1997	USA/Florida	FG	-81.52	27.66	North America	24.24	-90.12
DQ631892	2005	Mexico	FG	-107.39	24.8	North America	24.24	-90.12
EF110890	2006	USA/Texas	FG	-99.9	31.97	North America	24.24	-90.12
FJ609655	2006	Mexico	FG	-108.82	25.76	North America	24.24	-90.12
FJ012358	2006	Mexico	FG	-108.46	25.56	North America	24.24	-90.12
AJ223505	1997	Cuba	FG	-77,78	21,52	North America	24.24	-90.12
AF024715	1994	Dominic	FG	-70,16	18,74	North America	24.24	-90.12
AJ865337	1997	Reunion	FG	55.48	-21.33	Reunion	-21.19	55.37
AM409201	2004	Reunion	FG	55.26	-21.06	Reunion	-21.19	55.37
AY044138	1996	Sudan	CP	33.5	14.5	Horn of Africa	11.45	36.41
EF051116	2005	Lebanon	CP	35.86	33.85	E Mediterranean	33.11	33.02
EF054893	2005	Jordan	CP	36.24	30.59	E Mediterranean	33.11	33.02
EF054894	2005	Jordan	CP	36.24	30.59	E Mediterranean	33.11	33.02
EF107520	1991	Egypt	CP	29.78	30.78	E Mediterranean	33.11	33.02
EF158044	2005	Jordan	CP	36.24	30.59	E Mediterranean	33.11	33.02
EF185318	2005	Lebanon	CP	35.86	33.85	E Mediterranean	33.11	33.02
FJ030876	2005	Egypt	CP	31.21	30.08	E Mediterranean	33.11	33.02
GQ861426	2008	Jordan	CP	36.24	30.59	E Mediterranean	33.11	33.02
GQ861427	2008	Jordan	CP	36.24	30.59	E Mediterranean	33.11	33.02
X15656	1988	Israel	CP	34.85	31.05	E Mediterranean	33.11	33.02
X76319	1993	Israel	CP	34.85	31.05	E Mediterranean	33.11	33.02
AJ812277	2004	Turkey	CP	34.63	36.8	E Mediterranean	33.12	33.03
AJ867486	2004	Turkey	CP	34.63	36.8	E Mediterranean	33.13	33.04
AJ867487	2004	Turkey	CP	28.37	37.22	E Mediterranean	33.14	33.05
AM691085	2006	Greece	CP	27.97	36.16	E Mediterranean	33.16	33.07
AM691759	2006	Greece	CP	28	36.3	E Mediterranean	33.17	33.08
AB192965	2004	Japan	CP	133.4	33.4	Asia	28.17	121.40
AB192966	2004	Japan	CP	133.4	33.5	Asia	28.17	121.40
AB363566	2007	Japan	CP	127.82	26.37	Asia	28.17	121.40
AM282874	2006	China/Shanghai	CP	121.48	31.22	Asia	28.17	121.40
AM698117	2006	China/Zhejiang	CP	118	31	Asia	28.17	121.40
AM698118	2006	China/Zhejiang	CP	118	31	Asia	28.17	121.40
AM698119	2006	China/Zhejiang	CP	118	31	Asia	28.17	121.40
FN252890	2007	China/Zhejiang	CP	120.2	30.3	Asia	28.17	121.40
FN256256	2007	China/Zhejiang	CP	120.2	30.3	Asia	28.17	121.40
FN256257	2008	China/Shandong	CP	116	35	Asia	28.17	121.40
FN256258	2007	China/Shanghai	CP	121	31	Asia	28.17	121.40
FN256259	2008	China/Jiangsu	CP	118	32	Asia	28.17	121.40
GQ141873	2009	South Korea	CP	129.08	35.18	Asia	28.17	121.40
FJ646611	2008	China	CP	104.2	35.86	Asia	28.18	121.41
AF071228	1997	Spain	CP	-2.69	36.75	W Mediterranean	36.22	0.45
AF105975	1995	Portugal	CP	-7.93	37.02	W Mediterranean	36.22	0.45
AJ489258	1999	Spain	CP	-2.81	36.78	W Mediterranean	36.22	0.45
AJ519441	1999	Spain	CP	-2.81	36.78	W Mediterranean	36.22	0.45
DQ144621	2004	Italy/Sicily	CP	14.73	36.93	W Mediterranean	36.22	0.45
EF060196	2005	Morocco	CP	-2.33	34.92	W Mediterranean	36.22	0.45
EF101929	2005	Tunisia	CP	10.18	36.78	W Mediterranean	36.22	0.45
FJ439569	2008	Netherlands	CP	5.29	52.13	W Mediterranean	36.22	0.45
DQ644565	2004	Oman	CP	55.92	21.51	Iran	29.83	54.85
AJ132711	1996	Iran	CP	60.86	29.51	Iran	29.84	54.86
DQ855468	2005	Iran	CP	51.89	28.2	Iran	29.84	54.86
DQ855469	2003	Iran	CP	59.6	36.29	Iran	29.84	54.86
DQ855470	2003	Iran	CP	59.11	33.81	Iran	29.84	54.86
DQ855471	2003	Iran	CP	54.22	32	Iran	29.84	54.86
DQ855472	2004	Iran	CP	55.13	27.13	Iran	29.84	54.86
DQ855473	2004	Iran	CP	57.73	28.67	Iran	29.84	54.86
EF199813	2005	Iran	CP	50.68	33.99	Iran	29.84	54.86
EF199814	2006	Iran	CP	50.24	30.59	Iran	29.84	54.86
EF199815	2006	Iran	CP	48.98	31.46	Iran	29.84	54.86
EF199816	2006	Iran	CP	48.98	31.46	Iran	29.84	54.86
EF199817	2006	Iran	CP	48.98	31.46	Iran	29.84	54.86
EF199818	2005	Iran	CP	51.65	35.32	Iran	29.84	54.86
EU635776	2005	Iran	CP	57.82	28.27	Iran	29.84	54.86
GU076449	2006	Iran	CP	57.02	30.23	Iran	29.84	54.86
GU076448	2006	Iran	CP	57.08	30.25	Iran	29.84	54.86
GU076451	2006	Iran	CP	57.18	27.57	Iran	29.84	54.86
GU076445	2007	Iran	CP	55.68	28.92	Iran	29.84	54.86
GU076454	2006	Iran	CP	50.58	29.58	Iran	29.84	54.86
GU076440	2006	Iran	CP	54.18	31.73	Iran	29.84	54.86
GU076453	2006	Iran	CP	57.73	28.64	Iran	29.84	54.86
GU076441	2006	Iran	CP	57.71	28.58	Iran	29.84	54.86
GU076442	2006	Iran	CP	57.05	27.06	Iran	29.84	54.86
GU076443	2006	Iran	CP	57.05	27.1	Iran	29.84	54.86
GU076444	2007	Iran	CP	52.53	29.58	Iran	29.84	54.86
GU076452	2006	Iran	CP	57.72	28.64	Iran	29.84	54.86

GU076446	2007	Iran	CP	52.49	29.59	Iran	29.84	54.86
GU076447	2007	Iran	CP	52.52	29.57	Iran	29.84	54.86
GU076450	2007	Iran	CP	57.72	28.62	Iran	29.84	54.86
AY530931	1997	USA/Florida	CP	-81.52	27.66	North America	27.15	-101.21
DQ631892	2005	Mexico	CP	-107.39	24.8	North America	27.15	-101.21
EF110890	2006	USA/Texas	CP	-99.9	31.97	North America	27.15	-101.21
FJ012358	2006	Mexico	CP	-108.46	25.56	North America	27.15	-101.21
FJ609655	2006	Mexico	CP	-108.82	25.76	North America	27.15	-101.21
AJ842306	2004	Reunion	CP	55.26	-21.06	Reunion	-16.99	55.34
AJ842307	2004	Reunion	CP	55.26	-21.06	Reunion	-16.99	55.34
AJ842308	2004	Reunion	CP	55.26	-21.06	Reunion	-16.99	55.34
AJ865337	1997	Reunion	CP	55.48	-21.33	Reunion	-16.99	55.34
AM234066	2004	Reunion	CP	55.26	-21.06	Reunion	-16.99	55.34
AM409201	2004	Reunion	CP	55.26	-21.06	Reunion	-16.99	55.34
AY134494	2001	Puerto Rico	CP	-66.93	17.97	West Indies	13.07	-62.18
AY319646	2001	Guadeloupe	CP	-62.07	17.00	West Indies	13.07	-62.18
FM163453	2007	Grenada	CP	-61.64	12.18	West Indies	13.07	-62.18
FM163454	2007	Grenada	CP	-61.64	12.18	West Indies	13.07	-62.18
FM163455	2007	Grenada	CP	-61.61	12.13	West Indies	13.07	-62.18
FM163456	2007	Grenada	CP	-61.61	12.13	West Indies	13.07	-62.18
FM163457	2007	Grenada	CP	-61.65	12.13	West Indies	13.07	-62.18
FM163458	2007	Grenada	CP	-61.73	12.03	West Indies	13.07	-62.18
FM163459	2007	Grenada	CP	-61.73	12.03	West Indies	13.07	-62.18
FM163462	2007	Grenada	CP	-61.72	12.02	West Indies	13.07	-62.18
FM163463	2007	Grenada	CP	-61.72	12.02	West Indies	13.07	-62.18
